# Machine learning-based prediction of N2 lymph node metastasis in non-small cell lung cancer

**DOI:** 10.1186/s12890-025-03921-5

**Published:** 2025-10-06

**Authors:** Eren Erdogdu, İlkay Öksüz, Salih Duman, Berker Ozkan, Sukru Mehmet Erturk, Doğu Vurallı Bakkaloğlu, Murat Kara, Alper Toker

**Affiliations:** 1 Department of Thoracic Surgery, Faculty of Medicine, Istanbul University, Istanbul, Turkey; 2 Department of Computer Engineering, Istanbul Technical University, Istanbul, Turkey; 3 Department of Radiology, Istanbul University, Istanbul, Turkey; 4 Department of Pathology, Istanbul University, Istanbul, Turkey; 5 Department of Cardiovascular and Thoracic Surgery, West Virginia University, Morgantown, WV USA

**Keywords:** Lung neoplasm, Artificial intelligent, Neoplasm staging, Accuracy, Neoplasm metastasis

## Abstract

**Background:**

Lung cancer is a leading cause of cancer-related mortality worldwide. Accurate staging of mediastinal lymph nodes is a crucial step in determining appropriate treatment approaches. Current noninvasive diagnostic methods do not provide sufficient accuracy to confidently decide on surgery without histological confirmation. Our study aimed to develop a artificial intelligence model for the precise prediction of N2 lymph node metastasis.

**Methods:**

We retrospectively analyzed 1489 patients who underwent standard cervical mediastinoscopy at our department, including 472 patients diagnosed with non-small cell lung cancer. We developed three distinct prediction models for N2 lymph node station metastasis: one using standard statistical analysis, another utilizing an image processing deep learning algorithm with thoracic CT, and the third employing various machine learning methods with clinicopathological and radiological data. We compared diagnostic accuracy, area under the curve (AUC), sensitivity, and specificity rates, as well as the F1-score of all models.

**Results:**

Linear discriminant analysis, quadratic discriminant analysis, Gaussian naive Bayes, and artificial neural networks all surpassed 90% accuracy. The linear support vector machine demonstrated the highest performance, with an accuracy of 95.7%, an AUC of 93.5%, and an F1-score of 92%, respectively and outperformed the logistic regression-based statistical model, which reached an accuracy of 90.6% and an AUC of 85.7%.

**Conclusion:**

Machine learning models outperformed standard statistical analysis models in predicting N2 lymph node metastasis. Implementing these machine learning prediction models might greatly improve the accuracy of mediastinal lymph node metastasis detection, thereby enhancing clinical decision making and patient outcomes.

## Introduction

Lung cancer is the leading cause of cancer-related death worldwide. Treatment options and patient survival depend heavily on the stage. Surgery is considered the preferred treatment option for early-stage lung cancer without nodal metastasis. However, upfront surgery is not recommended in cases where nodal involvement in N2 and N3 stations or distant metastasis is detected. Only a small percentage of patients with advanced-stage disease undergo surgery after neoadjuvant treatment. Hence, prompt and accurate detection of nodal and distant metastases plays a crucial role in determining the appropriate treatment for patients. The utilization of PET/CT, along with enhancements in CT and MRI technology, has notably increased the radiological detection rate of nodal and distant metastases. The sensitivity and specificity of PET/CT for detecting distant metastasis have been reported as 83% and 93% [1]. However, PET-CT exhibits limited diagnostic accuracy for nodal metastasis, with reported pooled sensitivity of 62% and specificity of 92 [2]. Therefore, guidelines recommend confirming the absence of mediastinal nodal metastasis via invasive or surgical techniques such as EBUS or mediastinoscopy prior to surgery, whether for early stage lung cancer or following neoadjuvant treatment [3].

The evolving artificial intelligence (AI) technology holds a significant promise for advancing diagnostic procedures in the medical field. Its capacity to solve complex problems and detect intricate correlations renders it a valuable entity. AI models have been successfully applied in various aspects of thoracic surgery, including diagnosis, survival prediction, complication risk assessment, and treatment planning [4]. In this study, we aimed to compare the diagnostic accuracy of the prediction models for mediastinal lymph node metastasis by utilizing AI algorithms using clinical and radiological data, thus potentially eliminating the need for invasive procedures.

## Materials and methods

### Study design and participation

**Fig. 1:**
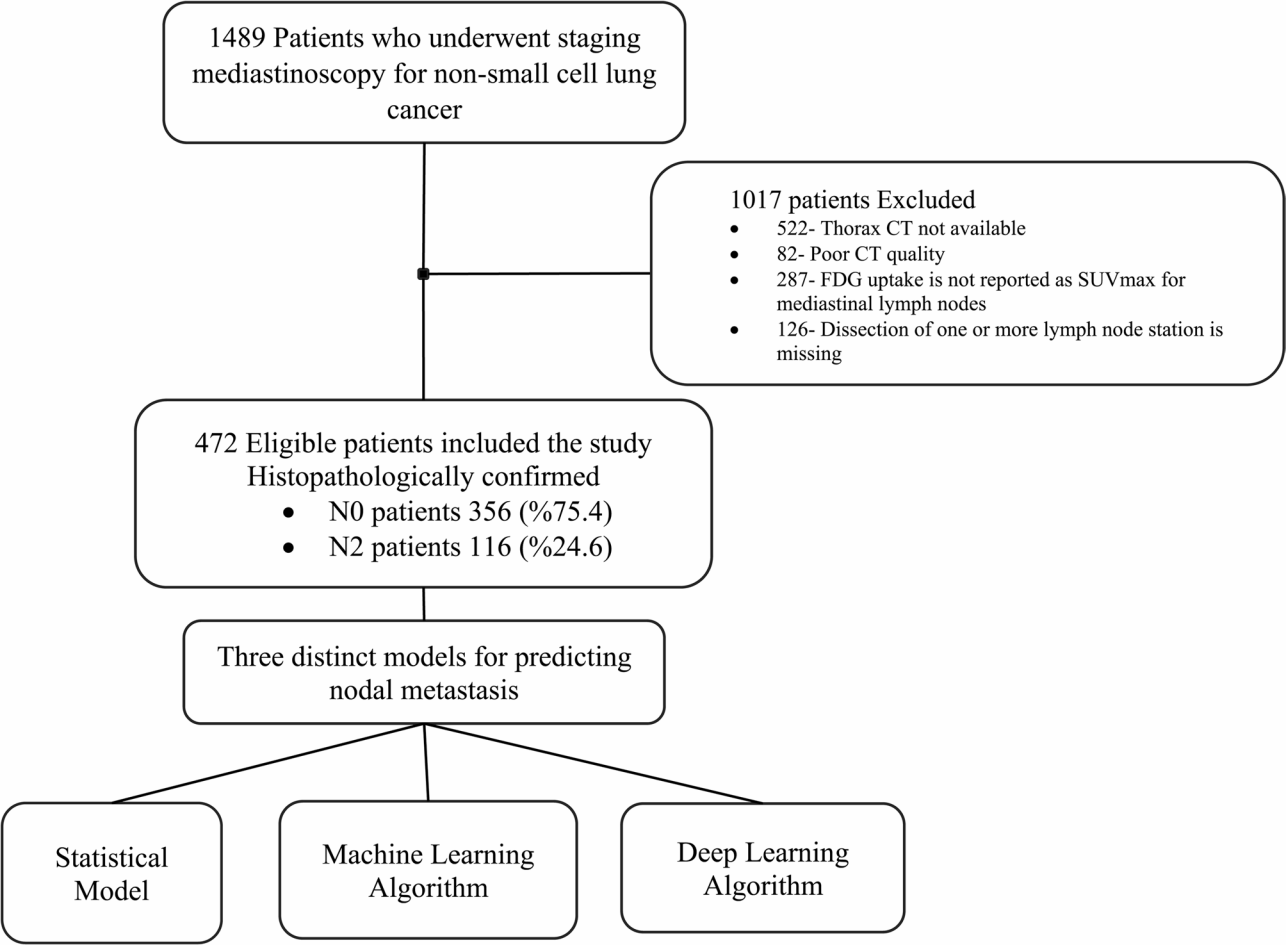
Flowchart of patient selection and modeling process

We retrospectively analyzed 1489 patients who underwent standard cervical mediastinoscopy at our institution between 2006 and 2021. We included patients with non-small cell lung cancer who underwent thoracic CT performed within two weeks before surgery and mediastinal lymph node dissection at stations 2R, 2 L, 4R, 4 L, and 7. Clinicopathological information, including age, sex, histopathological diagnosis, and radiological characteristics such as size and location of the tumor, was recorded. Additionally, FDG uptake of the tumor and lymph nodes in the bilateral upper paratracheal (2R, 2 L), bilateral lower paratracheal (stations 4R, 4 L), subcarinal (station 7), and tumor-side hilar (stations 10R or 10 L) regions were documented. Patients were excluded from the study if thorax CT was not available, CT quality was poor, FDG uptake was not reported as SUVmax for mediastinal lymph nodes, or dissection of any included lymph node station was missing (Fig. 1).

Mediastinoscopy indications were as described by ESTS consensus, including radiological or PET-CT suspicion of N2 involvement (short axis > 10 mm or SUVmax > 2.5), centrally located tumors, peripherally located tumors larger than 3 cm or tumors with high-risk histology. These criteria as described by ESTS guideline were applied at our institution throughout the study period [5].

**Fig. 2:**
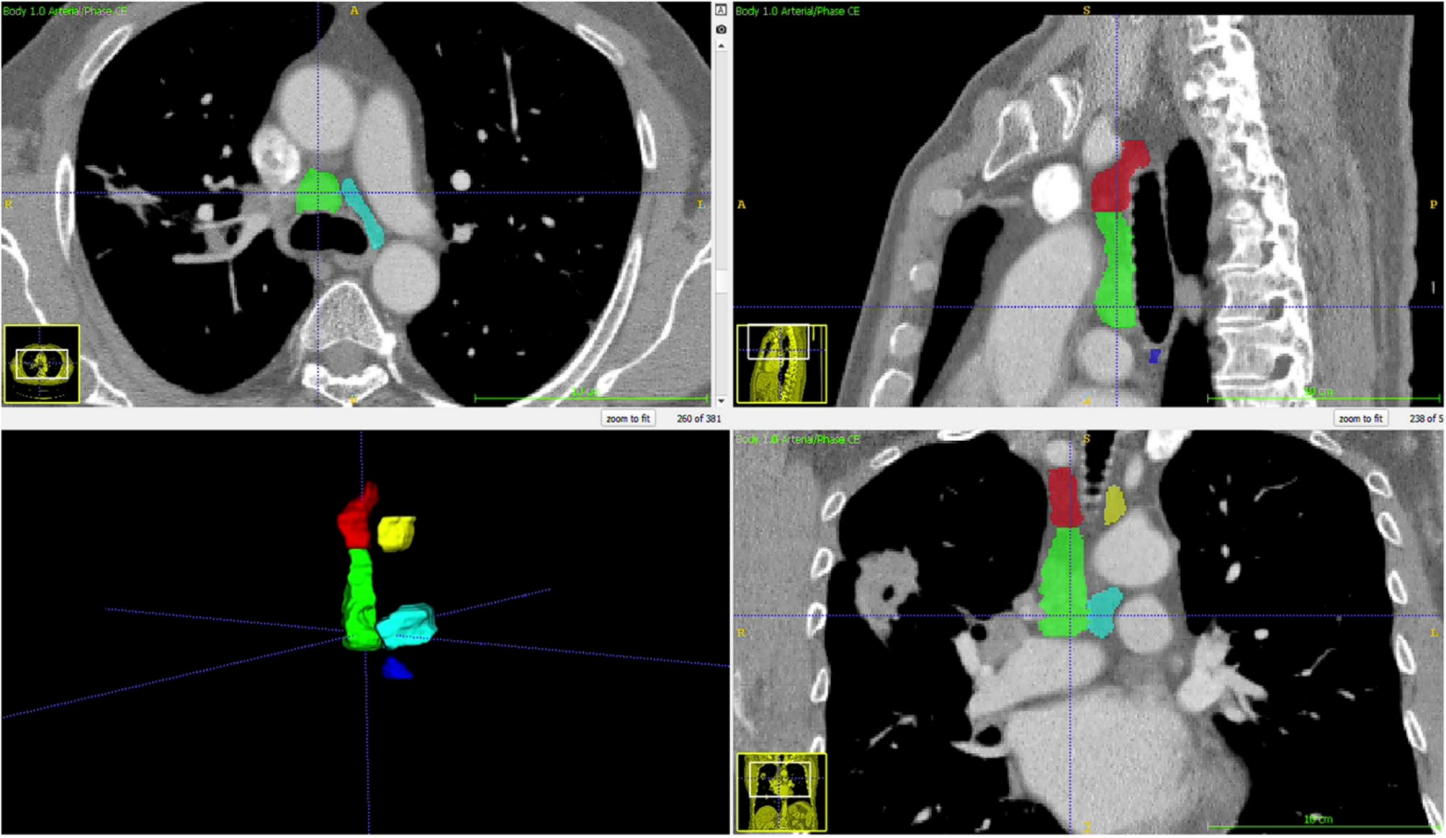
Example of 3D segmentation of the mediastinal N2 lymph node station

Thorax CTs were obtained in DICOM format and anonymized. A radiologist specializing in thoracic imaging measured the short and long axes of the largest lymph node at stations 2R, 2 L, 4R, 4 L, and 7 without clinical and pathological information. The radiologist also segmented these mediastinal N2 lymph node stations using ITK-Snap (version 3.8.0), and the segmentation files were stored as DICOM mask files in the NIFTI format (Fig. 2).

We divided the patients into two groups based on histopathologically confirmed N2 metastasis at the stations dissected using mediastinoscopy. Patients with metastasis in lymph node stations routinely accessed by standard cervical mediastinoscopy, deemed oncologically inoperable as N2 or N3, were included in the N2 group. Patients without metastasis at these stations, generally considered operable as N1 or N0, were assigned to the N0 group.

Operability of N2 disease was determined based on standard institutional practice. Single-station N2 disease was generally considered potentially operable, especially after neoadjuvant therapy. Multi-station or bulky N2 disease was classified as inoperable. In this study, we developed a prediction model for the presence of N2 metastasis regardless of operability status.

### Ethical standards

Our study was performed in accordance with the ethical standards of the 1964 Declaration of Helsinki and its later amendments or comparable ethical standards. This study was approved by the ethics committee of the Istanbul University Faculty of Medicine (No: 262880).

### Statistical analysis

Age, sex, tumor side, tumor location, adenocarcinoma subtype, radiological tumor size, FDG uptake of tumor, highest FDG uptake of N2 stations, tumor side hilar FDG uptake, FDG uptake ratio of tumor to highest N2 station, long and short axis of largest N2 lymph node, and time from PET/CT to surgery were all analyzed for N2 metastasis.

The Kolmogorov–Smirnov test was used to determine the distribution of continuous variables regarding N2 metastasis. Categorical variables were analyzed using the Chi-square and Fisher’s exact tests, as appropriate in the contingency tables. Similarly, Student’s t-test or Mann-Whitney U-test was performed to compare continuous variables, as appropriate. Categorical and continuous variables found to be statistically significant were analyzed using univariate regression. All the variables were also utilized in the machine learning algorithms. Stepwise logistic regression analysis was applied to univariate and multivariate analyses to confirm the impact of clinical and radiological factors on N2 metastasis. Using backward selection, we achieved a final reduced model by eliminating variables that were not statistically significant at *p* < 0.05. The importance of a diagnostic parameters in regression analysis reported with the hazard ratio and its 95% confidence interval. All statistical analyses were performed using the Statistical Package for Social Sciences (SPSS, version 25.0, Chicago, IL, USA).

### Development of machine learning algorithms

To develop the deep learning algorithm, we randomly divided the patients into three groups: 70% for training, 10% for validation, and 20% for testing. The raw DICOM data of thorax CT were adjusted to fit within a mediastinal window range of −140 to 260 Hounsfield Units, with regions outside the segmentation mask standardized to zero (Fig. 2). The algorithm was trained with only thoracic CT over 100 epochs using the ResNET50 architecture [6].

All clinical and radiological parameters listed in Table 1. were used as inputs for the machine learning algorithms, including linear support vector machine (SVM), linear discriminant analysis (LDA), quadratic discriminant analysis (QDA), Gaussian naive Bayes, AdaBoost, Random Forest, artificial neural network (ANN), and Gaussian process regression. The parameters included age, sex, tumor side (right or left), tumor lobe location (upper, middle, or lower lobe), histopathological subtype (adenocarcinoma or non-adenocarcinoma), radiological tumor size in millimeters, SUVmax of the primary tumor, the highest SUVmax of mediastinal N2 lymph nodes, SUVmax of the tumor-side hilar lymph node, the ratio of tumor SUVmax to the highest N2 station SUVmax, the long axis of the largest N2 lymph node in millimeters, the short axis of the largest N2 lymph node in millimeters, and the time interval from PET-CT to surgery in days. All machine learning models in this study were developed using Python, with the scikit-learn library (version 1.5.0), a widely recognized and extensively used open-source machine learning framework [7].

**Table 1.** Clinicopathological distribution of patients’ characteristics in terms of N2 metastasis

	**N (%)**	**Mean ± SD**	**N0**	**%**	**N2**	**%**	**p**
**Number of patients**	472 (100)		356	75.4	116	24.6	
Age		60.8 ± 9.1	61.2		59.5		0.431
Sex							
Female	85 (18)		54	63.5	31	36.5	
Male	387 (82)		302	78	85	22	0.005
Side							
Right	250 (53)		171	68.4	79	31.6	
Left	222 (47)		185	83.3	37	16.7	< 0.001
Tumor location							
Upper lobe	313 (66.3)		245	78.3	68	21.7	
Middle lobe	14 (3)		10	71.4	4	28.6	
Lower lobe	145 (30.7)		43	69.7	27	30.3	0.120
Adenocarcinoma subtype							
Adenocarcinoma	224 (47.5)		162	72	62	27	
Non-adenocarcinoma	248 (52.5)		194	78	54	22	0.130
Radiological tumor size (mm)		41.1 ± 20.6	41.7		39.1		0.140
SUVmax of the tumor		14 ± 7.4	13.8		14.7		0.090
Highest SUVmax of N2 station		2.4 ± 3.9	1.2		6.2		< 0.001
SUVmax of tumor side hilar station		2.1 ± 3.9	1.7		3.3		< 0.001
SUVmax Ratio of Tumor to highest N2 Station		1.5 ± 2.6	1.3		2		0.008
Long axis of largest N2 lymph node (mm)		15.7 ± 5.3	13.7		21.9		< 0.001
Short axis of largest N2 lymph node (mm)		10.7 ± 4.1	9		15.8		< 0.001
Time from PET-CT to surgery (days)		65.7 ± 22.8	53.8		69.6		0.057

*SD* Standard deviation, *SUVmax* Maximum standardized uptake value

The Synthetic Minority Oversampling Technique (SMOTE) was used for normalization to mitigate the impact of the uneven distribution of N2 and N0 patients. Machine learning algorithms were executed with and without SMOTE [8]. We calculated the sensitivity, specificity, positive predictive value (PPV), negative predictive value (NPV), area under the curve (AUC), and accuracy (ACC) for both the model generated by statistical logistic regression analysis and all machine learning methods. We also calculated the F1 score, which is the harmonic mean of the precision and recall. Reported performance metrics (accuracy, AUC, F1 score, etc.) were derived from the test set. Lasso regression with five-fold cross-validation was applied using the scikit-learn library in Python to identify key predictive variables and address potential multicollinearity among the input features.

## Results

### Clinicopathological characteristics of patients

We included 472 patients according to the inclusion criteria (Fig. 1). Among them, 387 (82%) were male and 85 (18%) were female. The mean age was 60.8 ± 9.1 years. The tumor was located on the right side in 250 (53%) of the patients. In 313 (66.3%) cases, the upper lobes were the predominant tumor location. Adenocarcinoma was the most prevalent histopathological diagnosis, observed in 224 patients (47.5%), followed by squamous cell carcinoma in 205 patients (43.4%).

We detected N2 metastasis in 116 patients (24.6 %). Chi-square analysis revealed significantly more mediastinal lymph node (MLN) metastases in women **(***P =* 0.005) and right-sided tumors (*P <* 0.001). The lobe distribution showed a similar N2 metastasis rate (*P =* 0.120). Compared to other histopathological subtypes, adenocarcinoma did not significantly differ in terms of N2 metastasis (*P =* 0.130). Adenocarcinoma was significantly more prevalent among females (59 out of 85, 69.4%) than among males (165 out of 387, 42.6%) (*P <* 0.001). Moreover, nodal metastasis was significantly more common in females diagnosed with adenocarcinoma, occurring in 22 out of 59 cases (37.3%), versus 40 out of 165 cases (24.5%) in males (*P =* 0.040).

Our analysis revealed no significant difference in the mean radiological tumor size (*P =* 0.140) or the mean SUVmax of the primary tumor (*P =* 0.090) regarding N2 metastasis. Significant differences were observed between N0 and N2 cases in the highest SUVmax value of N2 station (*P <* 0.001), SUVmax value of tumor-side hilar lymph node (*P <* 0.001), and the SUVmax ratio of the tumor to the highest SUVmax value of N2 stations (*P=* 0.008).

In N2 patients, the short axis of the largest MLN was significantly greater, measuring 13.7 mm, compared to N0 patients, measuring 9 mm (*P <* 0.001). Similarly, the long axis was also significantly greater, measuring 21.9 mm compared to 13.7 mm in N0 patients (*P <* 0.001). The mean time interval between PET-CT and surgery was shorter in N0 patients (53.8 vs. 69.6 days), approaching statistical significance (*P =* 0.057) (Table 1.).

### Regression analysis

Female sex (*P =* 0.005), right-sided tumor (*P<* 0.001), highest FDG uptake of MLN station (*P <* 0.001), tumor-side hilar lymph node SUVmax (*P <* 0.001), SUVmax ratio of tumor to the highest SUVmax value of N2 stations (*P =* 0.022), long axis of the largest MLN (*P <* 0.010), and short axis of the largest MLN (*P <* 0.001) were associated with N2 metastasis in univariate regression analysis. However, multivariate regression analysis revealed that only female sex (*P =* 0.001), highest SUVmax of MNL stations (*P =* 0.003), long axis (*P =* 0.003) and short axis (*P <* 0.001) of the largest MLN station were associated with N2 metastasis (Table 2). The statistical logistic regression model predicted N2 metastasis with an accuracy of 90.7%, an AUC of 85.7%, and an F1-score of 80% (Table 3).

**Table 2:** Univariate and multivariate analysis of parameters by logistic regression

	Univariate			Multivariate		
	HR	% 95 CI	*p*	HR	% 95 CI	*p*
Sex (female vs. male)	2.04	1.233–3.373	0.005	4.969	1.949–12.664	0.001
Side (right vs. left)	2.31	1.484–3.595	< 0.001	1.064	0.508–2.229	0.860
Maximum FDG uptake of N2 station	1.467	1.351–1.592	< 0.001	1.225	1.070–1.402	0.003
Maximum FDG uptake of tumor-side hilar station	1.099	1.044–1.156	< 0.001	1.003	0.897–1.121	0.960
FDG Uptake Ratio of Tumor to highest N2 Station	1.091	1.013–1.174	0.020	1.002	0.877–1.144	0.970
Long axis of largest N2 lymph node	1.54	1.418–1.672	< 0.001	1.200	1.064–1.352	0.003
Short axis of largest N2 lymph node	1.979	1.74–2.25	< 0.001	1.569	0.508–2.229	< 0.001

**Table 3:** Comparison of predictive models for detecting N2 lymph node metastasis

Model	Accuracy	AUC	Sensitivity	Specificity	PPV	NPV	F1-score
SPSS Logistic Regression	0.906	0.857	0.759	0.955	0.846	0.923	0.8
Linear SVM^a^	0.957	0.935	0.885	0.985	0.958	0.957	0.92
Linear SVM (SMOTE^b^)	0.904	0.91	0.923	0.897	0.774	0.968	0.842
Radial basis function SVM	0.787	0.615	0.231	1.000	1.000	0.773	0.375
Radial basis function SVM (SMOTE)	0.894	0.867	0.808	0.926	0.808	0.926	0.808
Linear discriminant analysis	0.926	0.877	0.769	0.985	0.952	0.918	0.851
Linear discriminant analysis (SMOTE)	0.926	0.913	0.885	0.941	0.852	0.955	0.868
Quadratic Discriminant Analysis	0.904	0.863	0.769	0.956	0.87	0.915	0.816
Quadratic Discriminant Analysis (SMOTE)	0.915	0.894	0.846	0.941	0.846	0.941	0.846
Decision Tree	0.904	0.874	0.808	0.941	0.84	0.928	0.824
Decision Tree (SMOTE)	0.883	0.86	0.808	0.912	0.778	0.925	0.792
Gaussian Naive Bayes	0.926	0.901	0.846	0.956	0.88	0.942	0.863
Gaussian Naive Bayes (SMOTE)	0.894	0.879	0.846	0.912	0.786	0.939	0.815
AdaBoost	0.894	0.867	0.808	0.926	0.808	0.926	0.808
AdaBoost (SMOTE)	0.872	0.852	0.808	0.897	0.75	0.924	0.778
Random Forest	0.883	0.8	0.615	0.985	0.941	0.87	0.744
Random Forest (SMOTE)	0.872	0.876	0.885	0.868	0.719	0.952	0.793
Artificial Neural Network	0.904	0.863	0.769	0.956	0.87	0.915	0.816
Artificial Neural Network (SMOTE)	0.915	0.906	0.885	0.926	0.821	0.955	0.852
Gaussian Process Regression	0.766	0.66	0.423	0.897	0.611	0.803	0.5
Gaussian Process Regression (SMOTE)	0.851	0.802	0.692	0.912	0.75	0.886	0.72

^a^ SVM: Support vector machine

^b^ Smote: Synthetic Minority Oversampling Technique

### Machine learning and deep learning algorithms

Among the machine learning algorithms, the Linear SVM achieved the highest accuracy of 95.7% and an F1 score of 0.92. LDA, the ANN and Gaussian Naive Bayes also demonstrated high accuracy and outperformed statistical logistic regression model, achieving 92.6%, 90.4%, and 92.6%, respectively. The application of the SMOTE algorithm improved the accuracy of the RBF SVM, QDA, ANN, and Gaussian Process Regression. The most significant difference was observed in RBF SVM with an increase of 13.5%, and in Gaussian Process Regression with an increase of 11% (Table 3). The deep learning image processing algorithm predicted all cases as metastasis-negative and failed to detect N2 metastases.

**Fig. 3:**
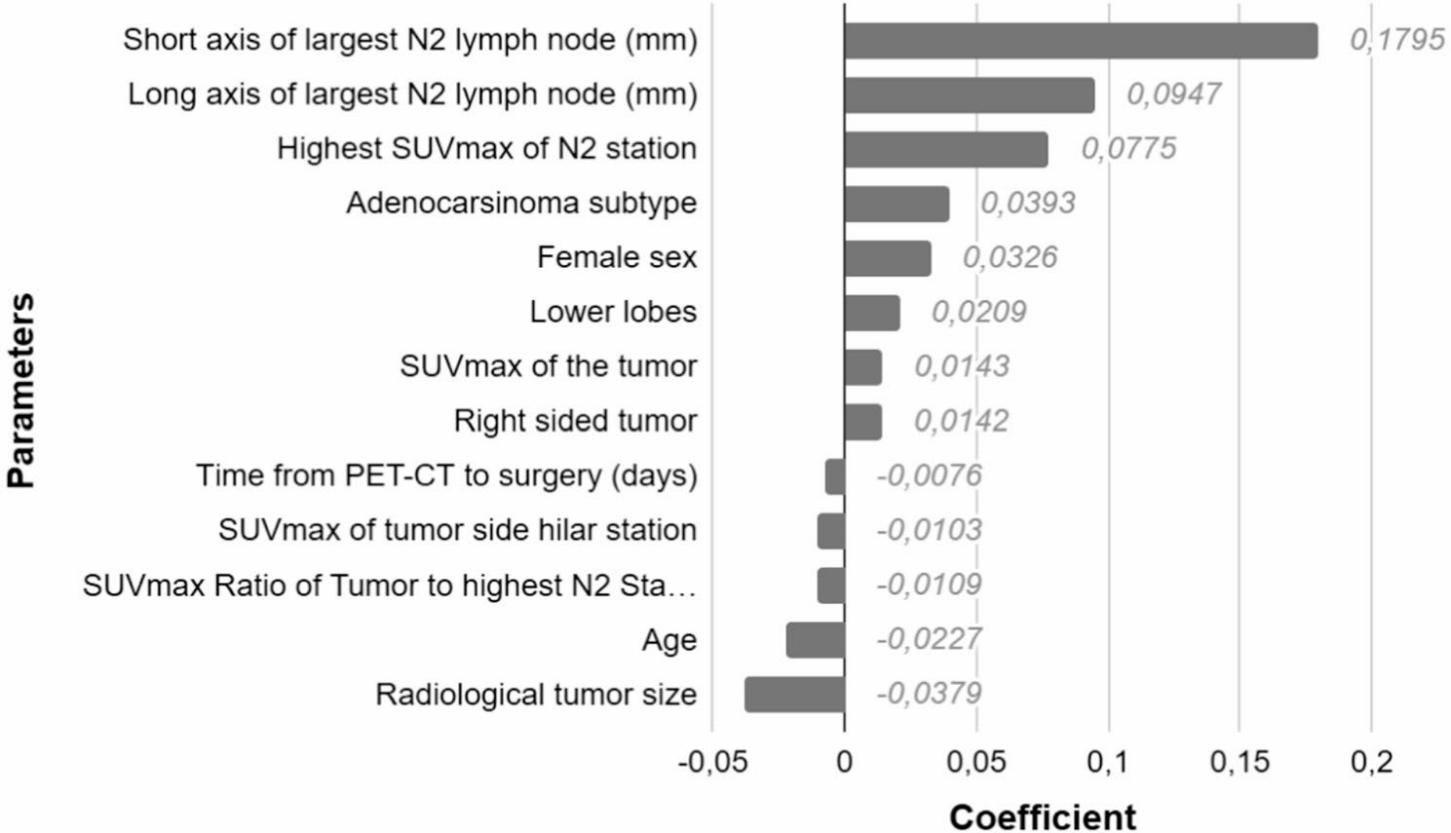
Lasso regression coefficients for variables predicting N2 lymph node metastasis

Lasso regression analysis identified several variables with non-zero coefficients. The highest coefficient was observed for the short axis of the largest N2 lymph node (0.1795), followed by the long axis (0.0947) and the highest SUVmax of the N2 station (0.0775). Other variables with positive coefficients included adenocarcinoma subtype (0.0393), female sex (0.0326), lower lobe tumor location (0.0209), SUVmax of the tumor (0.0143), and right-sided tumor (0.0142). Variables with negative coefficients included time from PET-CT to surgery (–0.0076), SUVmax of the tumor-side hilar station (–0.0103), SUVmax ratio of the tumor to the highest N2 station (–0.0109), age (–0.0227), and radiological tumor size (–0.0379) (Figure 3).

## Discussion

Our study demonstrated that machine learning can improve the accuracy of mediastinal lymph node metastasis prediction. This study presents several significant strengths. First, we employed a variety of machine-learning algorithms, allowing for a comprehensive comparison of their predictive capabilities. Notably, LDA, the ANN, and Gaussian Naive Bayes achieved an ACC rate over 90% and outperformed the traditional statistical model, underscoring the potential of machine learning for this topic. The Linear SVM model stands out, achieving an impressive accuracy rate exceeding 95%. Our research is distinguished by its substantial cohort size, which is among the largest in the literature for this specific application, thus enhancing the reliability and generalizability of our findings. Importantly, the ground truth for our model was based on pathological diagnosis, ensuring a high level of reliability of the results.

The implementation of machine learning algorithms may potentially reduce the need for mediastinal staging with an additional invasive diagnostic procedure, as recommended by the NCCN guidelines for NSCLC. This approach aims to exclude possible occult N2 disease, which literature reports have identified in 6.5-16.8% of clinical N0 cases [9] and in 14-16% of clinical N0/N1 cases [10]. Therefore, clinical N0 patients should be meticulously investigated for N2 disease. While non-surgical invasive diagnostic methods, such as EBUS/EUS, are commonly used, these procedures increase time, costs, and procedural risks and may not always be necessary for treatment decisions. Moreover, a specificity of 95.7%, which accomplished by Linear SVM, is crucial for ruling out false-positive results of MLN metastasis on PET/CT, particularly in tuberculosis endemic countries, where PET/CT shows lower sensitivity and specificity[2]. 

In the creation of statistical and machine learning models, we used the commonly available parameters, which are reported to be related to nodal metastasis. While young age has been reported as a high-risk factor for nodal metastasis [11], our analysis did not find this association to be significant. Sex was not considered as a risk factor for MLN metastasis. However, the multivariate regression analysis revealed a significant correlation. This may be attributed to the statistically significant higher prevalence of the adenocarcinoma subtype among females in the cohort, coupled with the higher incidence of N2 metastasis in patients with adenocarcinoma. Globally, the rate of lung cancers in females, particularly of the adenocarcinoma subtype, has been reported to be increasing[12]. Additionally, the rate of nodal metastasis in adenocarcinomas is reported to be higher than that in other histopathological types[13]. Our study found no correlation between the SUVmax of the primary tumor and nodal metastasis. However, Miyazaka et al. reported significantly higher SUVmax values for primary tumors in clinical N0 patients with occult N2 metastasis[14]. There were no significant differences in the tumor size. This contrasts with findings by Vaghini et al., who reported that each 1 cm increase in tumor size doubled the risk of nodal metastasis [15].

The first study on MLN metastasis prediction using machine learning was conducted by Vessele et al. in 2003 with 133 patients. They used an artificial neural network algorithm with only PET/CT parameters. Although the overall accuracy of predicting N status resulted was 87.3 %, the algorithm achieved a maximum accuracy of 94.9%, differentiated N0+N1 from N2+N3, and outperformed PET-CT reader, similar to our result[16]. Guotao Yin et al. reported ACC of 91.8% prediction N2 metastasis with a smaller cohort and only with SUVmax values, short and long axes, and densities of lymph nodes. Similar to our study, they reported that the SVM model was superior to the k-nearest neighbor and random forest mode[17]. In another study conducted by Gao et al. in 2021 with 132 patients, they investigated the SVM model using SUVmax values of mediastinal lymph nodes, short axes, texture analysis of lymph nodes in PET/CT images, and histogram data. They reported that the addition of texture analysis and histogram data to conventional radiological findings increased the sensitivity and specificity. Although the sensitivity rate was 96%, the specificity was limited to 75% and ACC rate was 86% [18]. This study is important in terms of showing that increasing the number of relevant variables is a contributing factor in the diagnosis of nodal metastasis.

Our study utilized a patient-based approach to categorize patients according to the presence or absence of nodal metastases. An alternative method is station-based labeling, in which each lymph node station is evaluated as an individual case. In a study in 2017, Wang et al. analyzed 1397 lymph nodes of 168 patients and compared thoracic CT tissue analysis and image processing methods with the radiological and clinical information of patients, as in our study. The same authors reported ACC, sensitivity, and specificity rates of the SVM model as 82.7%, 77.8%, and 88.3%, respectively, which were lower than our study[19]. Another station-based study by Pak et al. reported the results of a study in which 301 lymph nodes of 115 patients were evaluated separately. In this study, ACC was 40% and specificity was 96.2% with a decision tree model based on PET-CT findings, calcification, long and short diameters of lymph nodes[20]. Utilizing similar variables and with patient-based analysis, we found the same values of 90.4% and 94.1% in the decision tree model, respectively. The varying shapes, sizes, and characteristics of each lymph node station necessitate a comprehensive evaluation of the patient. Assessing each station separately may have led to a decrease in the overall accuracy.

We included one of the largest patient cohorts reported in the literature. Given the recent rise in the popularity of machine learning algorithms for the prediction of lymph node metastasis in other cancers and meta-analyses have been published[21, 22], only a few studies have reached approximately 500 patients[23, 24]. Although larger datasets typically enhance the performance of machine learning algorithms, feature selection remains crucial. In a multicenter study by Zhong et al. involving 3096 patients, a different approach was used to create a prediction model. They based the model solely on the patients' clinicopathological characteristics and radiological features of the primary nodule, without incorporating any CT or PET/CT variables related to mediastinal lymph nodes. Despite the large sample size, this approach resulted in a relatively low accuracy of 81%. Similarly, our image processing deep learning algorithm failed to detect MLN metastasis using only thoracic CT images without FDG uptake. The failure of our deep-learning algorithm may be attributed to several other factors. First, most of the cohort belonged to the N0 group, leading to a class imbalance that caused the algorithm to predict negative outcomes predominantly in the test group. Second, the model may be underfitting, either because the problem is more complex than anticipated or because thoracic CT images do not provide sufficient information to accurately detect N2 metastasis. Machine learning studies have demonstrated the effectiveness of PET/CT data in tumor staging, in a manner consistent with its utility in clinical practice.

The ground truth for the algorithm used in our study was based on histopathological diagnoses. Relying solely on radiological diagnoses for machine learning data carries the risk of replicating physician interpretations. Even with multiple physicians involved in the training and test groups, the algorithm tends to replicate the reference physicians’ judgment. In a study conducted by Wallis et al. in 2022 with 134 patients, three-dimensional lymph nodes extracted from PET-CT images were evaluated using a convolutional neural network. In this study, in which the results of the deep learning method were compared with the evaluations of two nuclear medicine specialists, it was observed that deep learning identified metastases with 88% sensitivity and 69% false positivity[25]. Another notable aspect of their study was the automated segmentation of the mediastinal stations. In contrast, our study employed the manual creation of masks to segment the MLN stations. Although automated segmentation algorithms for MLN are practical for daily clinical use, this technology is still evolving. Automated methods introduce a secondary consideration regarding the reliability of the results, specifically, in terms of accurately selecting the appropriate region of interest.

The limitations of our study include its retrospective design and specific cohort of patients who were referred for surgery and underwent mediastinoscopy. This decreased the occurrence of nodal metastasis and made our algorithms usable only for similar cohorts. Most machine-learning algorithms show a higher NPV and specificity. This was mainly a result of the low preoperative likelihood of MLN metastasis in the cohort. However, it is important to detect occult metastases in the upfront surgery group. Our cohort spans from 2006 to 2021, during which CT scanner technologies and protocols evolved. However, since our machine learning model primarily used measurably variables, the impact of scanner variability was minimized. Additionally, external validation using an independent cohort was not performed and should be addressed in future studies. Another limitation is that some variables used in the model may not be routinely available in all centers, particularly in resource-limited centers. Simplified models using fewer inputs could be developed in future research.

In conclusion, machine learning models created with clinicopathological and radiological data predicted mediastinal lymph node metastasis in NSCLC with high accuracy. Implementing these prediction models might contribute to clinical decision-making, leading to more precise treatment strategies and improved outcomes in patients with lung cancer.

## Data Availability

The data and algorithm used in this article will be available upon request from the corresponding author after publication if researchers submit a methodologically sound proposal aligned with the aims of the approved study.
